# Commensurate distances and similar motifs in genetic congruence and protein interaction networks in yeast

**DOI:** 10.1186/1471-2105-6-270

**Published:** 2005-11-09

**Authors:** Ping Ye, Brian D Peyser, Forrest A Spencer, Joel S Bader

**Affiliations:** 1 The Department of Biomedical Engineering, The Johns Hopkins University, Baltimore, MD 21218, USA; 2 The High-Throughput Biology Center, The Johns Hopkins University School of Medicine, Baltimore, MD 21205, USA; 3 The McKusick-Nathans Institute of Genetic Medicine, The Johns Hopkins University School of Medicine, Baltimore, MD 21205, USA; 4 The Department of Molecular Biology and Genetics, The Johns Hopkins University School of Medicine, Baltimore, MD 21205, USA

## Abstract

**Background:**

In a genetic interaction, the phenotype of a double mutant differs from the combined phenotypes of the underlying single mutants. When the single mutants have no growth defect, but the double mutant is lethal or exhibits slow growth, the interaction is termed synthetic lethality or synthetic fitness. These genetic interactions reveal gene redundancy and compensating pathways. Recently available large-scale data sets of genetic interactions and protein interactions in *Saccharomyces cerevisiae *provide a unique opportunity to elucidate the topological structure of biological pathways and how genes function in these pathways.

**Results:**

We have defined congruent genes as pairs of genes with similar sets of genetic interaction partners and constructed a genetic congruence network by linking congruent genes. By comparing path lengths in three types of networks (genetic interaction, genetic congruence, and protein interaction), we discovered that high genetic congruence not only exhibits correlation with direct protein interaction linkage but also exhibits commensurate distance with the protein interaction network. However, consistent distances were not observed between genetic and protein interaction networks. We also demonstrated that congruence and protein networks are enriched with motifs that indicate network transitivity, while the genetic network has both transitive (triangle) and intransitive (square) types of motifs. These results suggest that robustness of yeast cells to gene deletions is due in part to two complementary pathways (square motif) or three complementary pathways, any two of which are required for viability (triangle motif).

**Conclusion:**

Genetic congruence is superior to genetic interaction in prediction of protein interactions and function associations. Genetically interacting pairs usually belong to parallel compensatory pathways, which can generate transitive motifs (any two of three pathways needed) or intransitive motifs (either of two pathways needed).

## Background

A powerful tool to dissect the genetic buffering contributing to robustness of an organism is to identify gene pairs whose individual mutants are viable, but whose double mutants are lethal or exhibit reduced fitness [1,2]. These are particular types of genetic interactions, which more generally indicate that the phenotype of a double mutant differs from that expected from the phenotypes of the single mutants. Other types of genetic interaction include epistasis (an anticipated combined effect is not observed) and suppression (a defect is rectified by a second mutation). For convenience, we use genetic interaction henceforth to refer specifically to synthetic lethal and synthetic fitness genetic interactions.

Genetic interaction partners have been described as acting either in parallel compensating pathways, or in the same essential process [2]. Through revealing gene redundancy and compensating pathways, genetic interaction has contributed to the understanding of gene functions as well as the networks and pathways in which gene products participate [3-6]. It is also highly relevant to understanding genetic instability and variation occurring in various human diseases [2].

While a genetic interaction indicates that genes have compensating function, it does not necessarily indicate that the gene products work in the same pathway, for example as indicated by biochemical, physical interactions between proteins. Protein interactions can indicate correct network topology by linking proteins within the same biological pathway. The recent availability of high-throughput genetic interaction screens [3-6] and protein interaction screens [7-10] for the model organism *Saccharomyces cerevisiae *(budding yeast) provides a unique opportunity to investigate the genetic interaction network and protein interaction network both individually and jointly. Genetic interactions often reflect functional relationships that reach far beyond local protein interactions. Protein interaction data from high-throughput approaches are known to include false positive as well as physiologically relevant observations. It is critical to understand the correlations between genetic and protein interactions, as information derived from these two types of networks can provide complementary views for developing our understanding of how genes function in specific biological pathways, and how failures of these pathways lead to pathologic conditions that are relevant to the occurrence and progression of human diseases.

Graph theoretic approaches have been applied to study global properties of protein interaction networks and genetic interaction networks in yeast [6,11-22]. A few global network analyses also directly compared the genetic and protein interaction maps. It has been suggested that the current genetic interaction network is at least four times denser than the protein interaction network; genetic interactions are significantly more abundant between physically interacting proteins and the number of common genetic neighbors between two genes correlates with a known protein-protein interaction [6]. Other studies show that highly connected hubs in the protein network have a higher probability to genetically interact with each other [23], that the two-hop physical-genetic interaction is the top predictor of genetic interactions [24], and that probabilistic network models favor between-pathway explanations over within-pathway explanations for synthetic lethal genetic interactions [22].

Here, we present a global and local network investigation of the connections among genetic interaction, genetic congruence, and protein interaction networks for yeast, focusing on quantitative comparison of path length and motifs. Our results demonstrate that the genetic congruence network inferred from direct genetic interactions largely overlaps with the protein interaction network, with corresponding distances and motifs, while the genetic interaction network does not. This finding indicates that genetic congruence provides evidence for physical interaction and protein complex membership, as well as similar gene functions. The genetic congruence network we have defined can function as a mini-map to reveal network properties before the entire genetic interaction map is completed in yeast.

## Results

### Network overview

**Figure 1:**
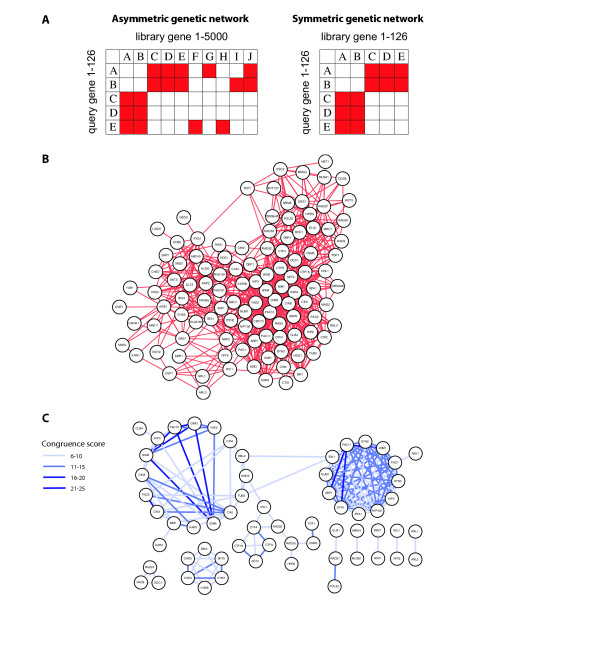
**Schematic illustration of genetic and congruence networks**. **A. **Asymmetric and symmetric genetic networks are represented in matrix form; filled squares represent observed genetic interactions. The symmetric network includes only genes used as queries. **B. **The symmetric genetic interaction network contains 126 genes. **C. **A congruence network was calculated from the symmetric genetic interaction network using a threshold congruence score of 6.

The genetic interactions used in this study are taken from published experiments detecting cell growth defects through screening a deletion of interest (query gene) against ~5000 viable yeast single-deletion strains (target genes) [3-6]. As only ~150 queries have been reported, the current network covers ~2% of the entire map. Therefore, many observations will be re-assessed after completion of the map. Specifically, the entire observed genetic network is expected to be *symmetric *when query and target genes are reversed. To account for the symmetric property of the entire genetic network, we have constructed both an *asymmetric genetic network *that includes all currently available high-throughput genetic interactions and the *symmetric genetic network *that covers interactions only between genes that have been used as queries (Fig. 1A). The graph of the symmetric genetic network is shown in Fig. 1B. Each node represents a gene, and each edge represents the genetic interaction between two connected genes. The edges are considered undirected, and we do not distinguish between edges that were detected in one or both directions. High connectivity in the symmetric genetic network (Fig. 1B) reflects that query genes were selected based on related functionality [6].

Previous analysis has suggested that shared genetic interaction partners correlate with physical interactions [6]. Quantitative measures for partner sharing in physical interaction networks has been defined as Mutual Clustering Coefficients (MCC) [14]. Here we use the negative Log_10 _of the P-value of the hypergeometric MCC as a quantitative measure of neighbor sharing in the genetic interaction network, and for convenience term it the congruence score [25]. Higher scores indicate that two genes share more genetic interaction partners than expected by chance. A genetic congruence network is then derived from introducing non-directed edges between congruent genes, using the congruence score to provide an edge weight (Fig. 1C). Asymmetric and symmetric congruence networks have been constructed from the corresponding genetic networks, respectively. A P-value of 0.01 for shared genetic interaction partners after correcting for multiple testing corresponds to a congruence score of 8 for the congruence network derived from the asymmetric genetic interactions and a congruence score of 6 for the network derived from the symmetric genetic interactions.

The protein interaction network we used is derived from ~45,000 protein-protein interactions compiled from the large-scale yeast two-hybrid and mass spectrometry analyses [7-10]. Each interaction has been assigned with a confidence score that corresponds to the network edge weight. The confidence score represents the probability that two proteins interact with each other [12].

The size and global topological measures for genetic, congruence, and protein networks are provided (Table 1). The average degree is the number of edges per node, and the clustering coefficient measures the interconnectivity around a node. Interestingly, average degrees nearly halve but clustering coefficients double from genetic networks to congruence networks. The values for the protein network are in between those for genetic and congruence networks. These suggest that the congruence network tends to be highly clustered. We quantitatively demonstrate with the following results on path lengths and local motifs that the inferred congruence links from shared patterns of genetic interactions have greater relevance to protein interactions than underlying direct genetic interactions.

**Table 1:** Standard global topological measures describing network structure. Detailed analyses on path lengths and local motifs are described in Fig. 2 and 3.

	Asymmetric genetic network	Symmetric genetic network	Asymmetric congruence network *Threshold = 8*	Symmetric congruence network *Threshold = 6*	Protein network *Threshold = 0.5*
No. of nodes	1004	111	122	61	3208
No. of edges	3799	813	267	146	13038
Average degree	7.6	14.6	4.4	4.8	8.1
Average clustering coefficient	0.10	0.37	0.73	0.84	0.45

### Network distances

Conventional analysis shows genetically interacting genes encode proteins in the same complex more often than would be expected by chance [6]. Because physical associations and genetic interactions each report on functional similarity, we might naively expect that physical and genetic links should be correlated. However, it has also been recognized that the number of genetic interaction pairs having direct physical interaction is a small fraction of the total number of genetic interaction pairs (~1%) [6]. Therefore, given currently known genetic and protein interactions and their overlap, the majority of genetic interactions do not connect physical partners.

**Figure 2:**
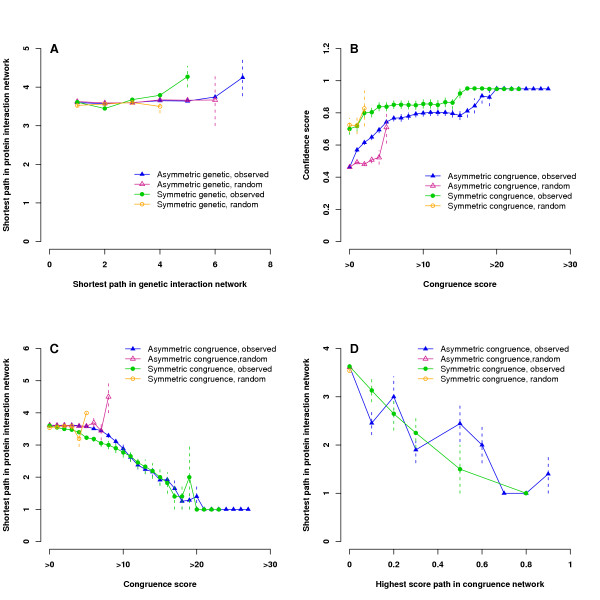
**Path length comparison for genetic, congruence, and protein networks**. **A. **There is little correlation between short paths in the genetic interaction network and short paths in the protein interaction network. **B. **Protein interaction confidence increases with congruence score. **C. **The path length in the protein network decreases monotonically with the congruence score. **D. **High-scoring paths in the congruence network are correlated with short distances in the protein interaction network, indicating that these networks are commensurate. Results are displayed for the observed and randomized networks. Error bars indicate one standard error. The random value if present is comparable to the observed value (P-value > 0.05).

To quantitatively study the global relationships between genetic and physical interactions, we calculated the shortest path length for any two genes in the genetic interaction network and the shortest path length for corresponding gene products in the protein interaction network, and then compared these two path lengths. Our results reveal that most protein pairs are distributed 3–4 links apart in the protein interaction network, regardless of whether there is a genetic interaction between the gene pair (Fig. 2A). This indicates that characteristic path lengths in genetic and physical interaction networks are incommensurate. Results are similar using symmetric and asymmetric genetic networks. These observations support the concept that genetically interacting pairs usually have no direct physical interactions. If we define pathways by the context of physical interactions and assume genes with physical interactions function in a single pathway and without physical interactions act in parallel pathways, then our results suggest that genetically interacting genes are more likely to belong to parallel compensating pathways. Other groups have used similar reasoning to identify components of pairs of complementary pathways from joint analysis of physical and genetic interactions [22].

We asked whether the other view of genetic interactions, i.e. genetic congruence, might yield improved concordance with physical interactions. We first explored the relationship between pair-wise genetic congruence versus direct physical interaction. High-throughput physical interaction data sets are known to include many false-positives, which can confound analysis. Confidence scores have been developed to reflect the probability that a physical interaction is a true-positive [12]. We observed that protein interaction confidence increases with the congruence score (Fig. 2B). Above the congruence score of 8 and 6, which corresponds to the network P-value of 0.01 for the asymmetric and symmetric networks respectively, all protein pairs exhibit high confidence interactions with confidence score greater than ~0.8. This implies that genetic congruence acts as an indication of high-confidence protein interactions. It is notable that information from a purely genetic experiment correlates well with information from a purely proteomic experiment. We also used receiver operating characteristic (ROC) curves to assess the relationship of congruence scores and physical interactions. ROC curves for asymmetric and symmetric congruence scores both climb rapidly away from the origin with high true positive rates and low false positive rates [see additional file 1, supp. fig. S2]. According to the area under the curve, the congruence score from the symmetric network performs better than the score from the asymmetric network, but at the cost of making fewer predictions. This is in agreement with the result from Fig. 2B that congruence scores of the symmetric network predict higher confidence physical interactions as compared with those of asymmetric network. The reason for the differences may be due to biased selection of query genes, as the symmetric network only contains query genes and all query genes were selected from a few related biological processes [6].

We further investigated the pair-wise congruence in the context of the protein interaction network. Our results show that the shortest path of physical interactions between congruent pairs decreases from ~3.6 links to 1 link (direct physical interaction) with increasing of congruence score (Fig. 2C). The path length transition begins when the congruence score increases beyond 8 and 6 for asymmetric and symmetric congruence networks, respectively. Once the score reaches 21 and 20 for asymmetric and symmetric networks, the congruent gene encoded proteins coincide with known direct physical interactions (4 pairs with congruence score = 21 in the asymmetric network and 1 pair with congruence score = 20 in the symmetric network).

Finally, to explore the connection between the congruence network and the protein network, we computed the highest score path for any two genes in the congruence network. Edge weights are in the range of 0 and 1 generated by applying a sigmoid function to the congruence scores (see Methods). The higher the path score, the higher probability two genes share similar genetic interaction partners. When comparing the highest path score in the congruence network with the shortest path length in the protein interaction network, we observed that the physical distance decreases monotonically from the average path length ~3.6 links to 1 (direct physical interaction) as the highest path score increases in both asymmetric and symmetric congruence networks (Fig. 2D). Therefore, *transitive *genetic congruence is commensurate with physical distances, which is similar to *direct *genetic congruence (Fig. 2C).

### Network motifs

Network motifs represent significantly recurrent patterns of simple interactions in complex networks [17]. Comparison of local structures in the network can help reveal the connections among superficially unrelated biological or social networks [18]. Additionally, the local structure of the network contributes to the understanding of global organization of the network [16]. To contrast the local structure of three types of networks, we counted the abundance of non-directed triads and tetrads in genetic, congruence, and protein networks. The random networks used to detect tetrads were generated to preserve the same triad counts as the real network [18].

**Figure 3:**
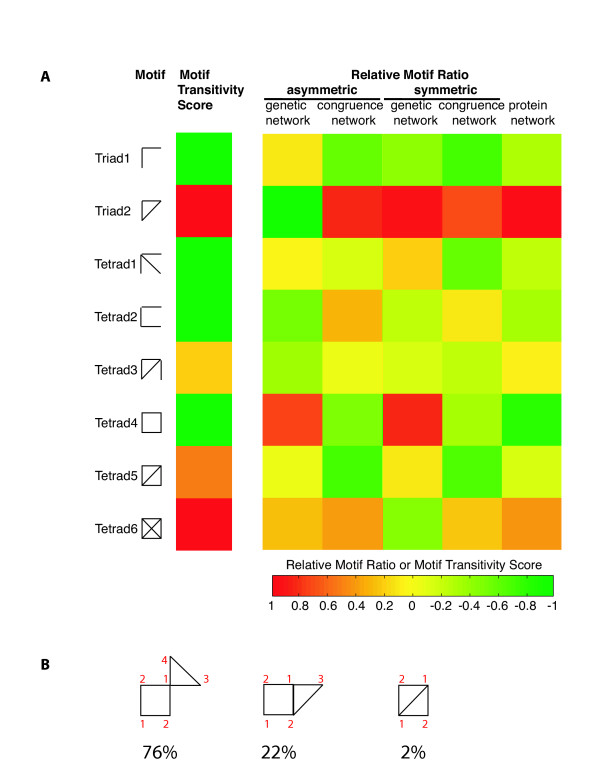
**Motif characterization for genetic, congruence, and protein networks**. **A. **Both transitive and intransitive motifs are enriched in the genetic network, tetrad4 and tetrad6 for the asymmetric network and triad2, tetrad1 and tetrad4 for the symmetric network. Only transitive motifs are enriched in congruence and protein networks, triad2 and tetrad6 for symmetric and asymmetric congruence networks, triad2, tetrad3, and tetrad6 for the protein network. Motif enrichment criteria are as defined in [17] (see Methods). **B. **The connections between triangle and square motifs in the symmetric genetic network. Three types of relationships exist between triangles and squares and the percentage of each scenario is labeled. The red numbers indicate individual pathways.

More significantly, we can determine network transitivity through the observation of whether a transitive or intransitive motif is enriched or depleted in the network. Transitivity is a common network property that interactions of A-B and B-C imply elevated probability of interaction of A-C. We developed a characteristic, termed the motif transitivity score (MTS), as a quantification of the motif transitivity [see Methods and additional file 1, supp. table S1]. The positive values indicate transitive motifs while the negatives represent intransitive motifs. The network transitivity has been quantified by the clustering coefficient before [26,27], which is closely related to the motif transitivity score defined here. We have found good agreement between motif enrichments (Fig. 3A) and average clustering coefficients (Table 1), i.e. congruence and protein networks are more clustered compared with the genetic network.

When using the asymmetric genetic and congruence networks for comparison with the physical network, the pattern of enriched motifs (the relative motif ratio) is significantly correlated for congruence and protein interaction networks (Pearson correlation coefficient R = 0.76, P-value = 0.03), and these are anti-correlated with the enriched motifs for direct genetic interactions (R = -0.66, P-value = 0.08; R = -0.69, P-value = 0.06, respectively) (Fig. 3A). This is consistent with the above global distance analysis, supporting significant overlap between congruence and protein networks.

The enriched motifs in asymmetric congruence and protein networks are all transitive, including triad2 (triangle motif) and tetrad6. The triangle motif is the most significantly enriched motif, suggesting the transitive property of congruence interactions and physical interactions. This result is in agreement with our observation in the previous section that the *transitive *congruence is correlated with short physical distance (Fig. 2D). The asymmetric genetic interaction network, however, consists of both intransitive motif tetrad4 (square motif) and transitive motif tetrad6, with the square motif as the most enriched structure.

The detection of intransitive motifs in the asymmetric genetic network may be due to the artifact that the interactions have not yet been tested. It does not necessarily mean that these interactions do not exist. To overcome this bias, we repeated motif-finding procedure using the symmetric genetic network and corresponding congruence network (Fig. 3A). The pattern of enriched motifs is still significantly correlated for symmetric genetic congruence and protein interaction networks (Pearson correlation coefficient R = 0.73, P-value = 0.04), but these are insignificantly correlated with those for the symmetric genetic network (R = 0.29, P-value = 0.49; R = 0.10, P-value = 0.82, respectively). The enriched motifs in the symmetric congruence network remain the same as for the asymmetric congruence network, i.e. all transitive motifs, triad2 (triangle motif) and tetrad6.

A final concern is that the transitive motifs arise from the mathematical process of generating congruence scores: if genes A and B share synthetic lethal partners, and B and C share partners, then A and C may have an increased probability of sharing partners. To address the question, we followed the following protocol [see additional file 1, supp. fig. S3]: (1) Randomize the genetic interaction network. (2) Calculate congruence scores for gene pairs in the randomized network. (3) Set a threshold and calculate motif enrichment for the random congruence network. We repeated this process 100 times for both the symmetric and the asymmetric genetic interaction networks. The typical extreme value for the maximum congruence score observed was 5 for the symmetric network and 6 for the asymmetric. Thus, applying the same cutoff for congruence scores as in the actual network, 6 for symmetric and 8 for symmetric, typically rejects all the congruence edges in the randomized network. We reduced the thresholds to retain the same number of congruence edges as in the actual network, with mean values of 1.8 (symmetric) and 3.2 (asymmetric) over the 100 randomizations. The average clustering coefficient is significantly smaller in the random networks than the actual network: 0.23 vs. 0.84 (random vs. actual symmetric, P-value 10^-402^), and 0.12 vs. 0.73 (random vs. actual asymmetric, P-value 10^-933^). Although the transitive motif triad2 (triangle) is enriched in the random congruence network relative to a fully random network, the motif count is far below that observed in the actual congruence network [see additional file 1, supp. table S3]. Other patterns of motif enrichment are quite different: tetrad4 (square motif, intransitive) is enriched in the random congruence network and depleted in the actual network, and tetrad6 (4-clique, transitive) is enriched in the actual network but not in the random network [see additional file 1, supp. fig. S4]. The transitive motifs in the congruence network are therefore enriched significantly beyond what would be expected based solely on the method of defining congruence edges.

Both transitive and intransitive motifs are still detected in the symmetric genetic interaction network. However, the types are different from those in the asymmetric genetic network. The transitive triangle motif becomes the most enriched structure in the symmetric genetic network, in agreement with a previous study that genetic interaction partners of a gene have an increased likelihood to interact with each other [24]. One source of the triangle motif could be the requirement for any two of three pathways for viability. Notably, the square motif is still highly enriched in the symmetric genetic network despite the abundance of the triangles, indicating that the square motif will remain enriched when the complete genetic interaction network is determined.

The view from recent studies indicates that high clustering is a generic feature of biological networks, as exemplified by protein interaction and protein domain networks [13]. However, we find that the genetic interaction network has both transitive and intransitive motifs. The coexistence of triangle and square motifs in the genetic network suggests two scenarios for genetic interactions between pathway components. In one scenario, genetic interactions between two pathways generate a square motif. Each edge crosses between the pathways, and genes at opposite corners are in the same pathway. In the second scenario, any two of three pathways are required for viability. Genetic interactions cross between all three pathways, generating the triangle motif.

To further answer the question whether the enriched triangles and squares overlap with each other or are excluded from each other, we compared the members of triangle and square motifs in the symmetric genetic network (Fig. 3B). Results show that one-node sharing is the dominant scenario (76%) for triangles and squares. Assuming three pathways for the triangle motif and two pathways for the square motif, the one-node sharing case defines four parallel pathways with one shared by the square and triangle. Two-node sharing accounts for 22% of total possibilities, and suggests three parallel pathways with two shared by the triangle and square. Only 2% of total cases are the complete overlap of the triangle and square, which is in an agreement with our observation that tetrad5 is not an enriched motif in the symmetric genetic network (Fig. 3A).

Because the completed genetic interaction map will necessarily be symmetric (except for false-positives or false-negatives), the enriched motifs in the symmetric genetic network are more relevant than the enriched motifs in the asymmetric genetic network.

### Biological relevance

Correct interpretation of the relationship between genetic and protein interactions enables interesting biological predictions. As we have demonstrated in previous sections, genetic congruence and protein networks are similarly organized with corresponding distances and motifs. Then, we would expect that two genes closer in the congruence network have higher tendency to physically interact with each other, reside within one protein complex, and involve in similar biological process.

**Figure 4:**
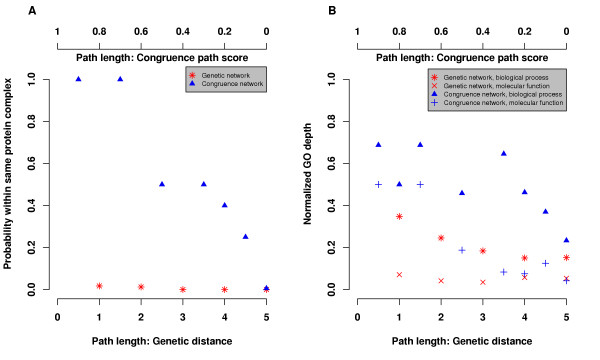
**Congruence network but not genetic network predicts protein complex membership and functional association**. **A. **Short distance in congruence network implies protein complex membership. B. Close distance in congruence network suggests similar function. GO [28] hierarchy depth is normalized to the range of 0 and 1 by [depth-min(depths)]/[max(depths)-min(depths)], where depths are calculated for each GO category, biological process and molecular function. As distance results are similar for symmetric and asymmetric networks, we only present those for the symmetric network.

To validate this prediction generally, we plotted protein complex membership versus the distance in the genetic network and the path score in the congruence network (Fig. 4A). The probability of co-residence in a protein complex increases with the congruence path score, and scores greater than 0.7 indicate same protein complex membership. On the other hand, gene products binned by distance in the genetic interaction network have uniformly low probability of protein complex co-residence.

Physical interactions usually suggest functional association. Accordingly, we asked whether congruence also indicates functional connection besides physical connection. As an initial validation, we found that genes close in the congruence network share similar functional annotations recorded in the database of Gene Ontology (GO)[28], i.e. biological process and molecular function (Fig. 4B). Moreover, the functional similarity is consistently higher for gene pairs based on path score in the congruence network than based on distance in the genetic network.

An example of congruence coinciding with protein interaction and function association is the prefoldin complex, which includes *PAC10*, *GIM3*, *GIM4*, *GIM5*, and *YKE2*. These five genes are clustered in the congruence network and the average path score between any two members of this complex is 0.51 (Fig. 1C). They are all chaperone proteins forming a complex, which promotes efficient protein folding [29,30].

## Discussion

We have demonstrated that high genetic congruence implies high probability of a physical interaction and short distance in the physical interaction network. Short distances in the congruence network (measured by a high path score), but not in the genetic network, are commensurate with distances in the protein network. To account for false-positives in the high throughput protein interaction datasets, parallel analyses were performed using a protein network with edges weighted according to interaction confidence [see additional file 1, supp. fig. S5], and the results were similar to those obtained from the un-weighted protein network. A guide to the figures showing path length comparisons among genetic, congruence, and protein networks is provided [see additional file 1, supp. table S4]. Local structure indicates similar transitive motif enrichment in congruence and protein networks, while the genetic network significantly consists of transitive as well as intransitive motifs. Both global distance analysis and local motif analysis demonstrate that the genetic congruence network possesses similar network transitivity to the protein network.

The similarity between congruence and protein networks and the dissimilarity between genetic and protein networks have yielded three interesting conclusions with biological significance. *First*, we have demonstrated that significant genetic congruence correlates strongly with protein complex membership and functional association. *Second*, genetically interacting pairs usually belong to compensatory pathways without direct physical interactions. *Finally*, the coexistence of triangles and squares in the genetic network indicates that robustness may be due to two pathways that compensate each other (squares), or three pathways any two of which are needed (triangles).

While the protein interaction and genetic congruence networks exhibit a high degree of similarity, we do not expect them to be identical because they are based on distinctly different experimental measures. The protein interaction network is based on protein binding constants in cellular extracts under selective precipitation conditions [7,8] or within cells through over-expression of tested proteins [9,10]. The congruence network is based on growth defects exhibited by cells lacking a pair of gene products cultured under standard conditions[6]. Thus, high congruence may not necessarily indicate a physical interaction. The concordance we observed between congruence and protein interaction network structures provides strong support for the argument that they both faithfully reflect biologically relevant network relationships.

The conclusions drawn from our study are limited by the current coverage of genetic and protein networks. This is especially true for the genetic network, which is at low coverage. Moreover, the current genetic network is biased by query gene selection. The ~150 query genes all have relative large numbers of interaction partners and related functionality[6]. As the coverage and symmetric property are increased, we expect that the average degree and average clustering coefficient will decline. Network distance results are robust in response to changes in genetic network symmetry and protein network edge weight. The symmetric genetic network has been used for motif counting and the relative motif ratio is insensitive to network size[18]. Therefore, we believe that our conclusions on network distances and motifs should continue to hold as the entire genetic interaction network is mapped.

## Conclusion

In summary, we have demonstrated that genetic congruence is superior to genetic interaction in predicting protein interactions and within-pathway functional associations. In contrast, genetic interaction pairs usually act in parallel compensatory pathways. Motif study indicates that genetic interactions bear both transitive and intransitive characters. Consideration of the symmetric property of a complete genetic interaction network is crucial to determination of motif enrichment for the genetic network.

## Methods

### Genetic interaction networks

The genetic interaction dataset is derived from a recent high throughput study in budding yeast [6]. The interaction is detected by cell growth defect through introducing a deletion of interest (query gene) into all viable yeast single-deletion strains (target gene). Interactions derived from 6 essential query genes, including *MYO2*, *SCC1*, *CDC2*, *CDC7*, *CDC42*, and *CDC45 *were removed in our study because phenotypes exhibited by conditional alleles of essential genes may include loss of function, unregulated function, and gain of function, while null alleles of nonessential genes are by definition solely loss of function mutations. Results and conclusions do not change, however, when these 6 essential genes are included in the analysis.

We constructed two types of genetic networks. The *asymmetric genetic network *includes currently available high throughput genetic interactions, i.e. 3799 genetic interactions between 126 non-essential query genes and 982 target genes. The *symmetric genetic network *only contains interactions between query genes, i.e. 813 genetic interactions between 108 non-essential query genes and 104 target genes that have been used as queries.

### Randomization of genetic interactions

Genetic interactions from the high throughput study [6] were reported as an interaction between the query gene and the target gene. A randomized network was generated by keeping the query gene list unchanged, randomly matching one of the target genes according to the probability of each target gene shown in the interaction list with replacement. Duplicate query-target pairs and self-interaction pairs, which are not possible in the experimental networks, were rejected during randomization. Results depict the average over 1000 randomizations.

### Genetic congruence networks

The congruence score was defined as -log_10 _[hypergeometric P - value (*x *≥ *k*_obs_], and hypergeometric P−value (x≥kobs)=∑x=kmin⁡(m,n)C(m,x)C(t−m,n−x)/C(t,n)
 MathType@MTEF@5@5@+=feaafiart1ev1aaatCvAUfKttLearuWrP9MDH5MBPbIqV92AaeXatLxBI9gBaebbnrfifHhDYfgasaacH8akY=wiFfYdH8Gipec8Eeeu0xXdbba9frFj0=OqFfea0dXdd9vqai=hGuQ8kuc9pgc9s8qqaq=dirpe0xb9q8qiLsFr0=vr0=vr0dc8meaabaqaciGacaGaaeqabaqabeGadaaakeaacqqGqbaucqGHsislcqqG2bGDcqqGHbqycqqGSbaBcqqG1bqDcqqGLbqzcqqGGaaicqGGOaakcqWG4baEcqGHLjYScqWGRbWAdaWgaaWcbaGaem4Ba8MaemOyaiMaem4CamhabeaakiabcMcaPiabg2da9maaqahabaGaem4qamKaeiikaGIaemyBa0MaeiilaWIaemiEaGNaeiykaKIaem4qamKaeiikaGIaemiDaqNaeyOeI0IaemyBa0MaeiilaWIaemOBa4MaeyOeI0IaemiEaGNaeiykaKIaei4la8Iaem4qamKaeiikaGIaemiDaqNaeiilaWIaemOBa4MaeiykaKcaleaacqWG4baEcqGH9aqpcqWGRbWAaeaacyGGTbqBcqGGPbqAcqGGUbGBcqGGOaakcqWGTbqBcqGGSaalcqWGUbGBcqGGPaqka0GaeyyeIuoaaaa@6AB8@, where two target genes having m and n genetic interaction partners share x partners from a list of t query genes, and *C(j,k) *is the binomial coefficient j!/k!(j-k)! [25]. Related measures have been used to analyze protein interaction networks to predict protein-protein interactions [14]. The congruence score is calculated for every target gene pair in the symmetric and asymmetric genetic networks. The symmetric and asymmetric congruence networks are derived from the corresponding genetic networks, respectively. The distribution of network size over different congruence scores is provided [see additional file 1, supp. fig. S1]. The congruence score of 8 (122 nodes with 267 edges) for asymmetric congruence network corresponds to the network P-value of 0.01 after correction for multiple testing of per-link P-value 0.01/982^2 ^= 10^-8^. Similarly, the congruence score of 6 (61 nodes with 146 edges) is the cutoff value for the symmetric congruence network.

### Protein interaction network

We used 47,783 protein-protein interactions with confidence scores [12] compiled from the large-scale two-hybrid data sets of protein-protein interactions [9,10] and mass spectrometry analysis of protein complexes [7,8]. The distribution of network size over different confidence scores is provided [see additional file 1, supp. fig. S1].

### Network distances

The shortest path distance was counted for any two nodes in the un-weighted genetic interaction and protein interaction networks. The shortest path length is the sum of lengths of individual linkage.

The SEEDY algorithm [31] was used to compute highest score path distance for the weighted genetic congruence and protein interaction networks. The highest score path is the path with the maximal value of the product of edge weights. Disconnected components are ignored for both shortest path and highest score path calculations.

The edge weight for the protein network is the confidence score (in the range of 0 and 1) [12]. The edge weight for the genetic congruence network is derived from a sigmoid function w=e(s−a)/b1+e(s−a)/b
 MathType@MTEF@5@5@+=feaafiart1ev1aaatCvAUfKttLearuWrP9MDH5MBPbIqV92AaeXatLxBI9gBaebbnrfifHhDYfgasaacH8akY=wiFfYdH8Gipec8Eeeu0xXdbba9frFj0=OqFfea0dXdd9vqai=hGuQ8kuc9pgc9s8qqaq=dirpe0xb9q8qiLsFr0=vr0=vr0dc8meaabaqaciGacaGaaeqabaqabeGadaaakeaacqWG3bWDcqGH9aqpdaWcaaqaaiabdwgaLnaaCaaaleqabaGaeiikaGIaem4CamNaeyOeI0IaemyyaeMaeiykaKIaei4la8IaemOyaigaaaGcbaGaeGymaeJaey4kaSIaemyzau2aaWbaaSqabeaacqGGOaakcqWGZbWCcqGHsislcqWGHbqycqGGPaqkcqGGVaWlcqWGIbGyaaaaaaaa@432F@ (in the range of 0 and 1), where *s *is the congruence score, *a *and *b *are parameters. The rationale of introducing the above sigmoid function is derived from the probability distribution of Pr(true positive|*s*) = Pr(protein interaction|*s*) as genes sharing genetic interaction partners usually exhibit physical association [6]. The parameters *a *= 15.9 and *b *= 1.6 are the best-fit values for the sigmoid function to form a smoothed interpolation of Pr(protein interaction|*s*) for the asymmetric congruence network [see additional file 1, supp. fig. S6]. Results were not sensitive to the choice of parameter values [see additional file 1, supp. fig. S7]. Similarly, *a *= 17.7 and *b *= 3.4 are the best-fit values for the symmetric congruence network.

### Network motifs

We used the mfinder1.1 – network motifs detection tool  to count non-directed triad and tetrad motifs in genetic interaction, genetic congruence, and protein interaction networks. Both symmetric and asymmetric genetic networks were used for motif searching. Motifs were also counted for the symmetric congruence network with cutoff value of 6, the asymmetric congruence network with cutoff value of 8, and the protein network with confidence score greater than 0.5 [12]. Motif results are insensitive to the threshold values for congruence and protein networks [see additional file 1, supp. table S2]. The Metropolis algorithm was used to conserve the number of triads in random networks for tetrad motif counting. The relative motif ratio (RMR) was calculated to represent the abundance of each motif relative to random networks in which each node has the same number of edges as the corresponding node in the real network. The formula for RMR is defined as RMR=Δi/(∑Δi2)1/2, Δi=Nreali−<Nrandi>Nreali+<Nrandi>+ε, and ε=4
 MathType@MTEF@5@5@+=feaafiart1ev1aaatCvAUfKttLearuWrP9MDH5MBPbIqV92AaeXatLxBI9gBaebbnrfifHhDYfgasaacH8akY=wiFfYdH8Gipec8Eeeu0xXdbba9frFj0=OqFfea0dXdd9vqai=hGuQ8kuc9pgc9s8qqaq=dirpe0xb9q8qiLsFr0=vr0=vr0dc8meaabaqaciGacaGaaeqabaqabeGadaaakeaacqWGsbGucqWGnbqtcqWGsbGucqGH9aqpcqqHuoardaWgaaWcbaGaemyAaKgabeaakiabc+caViabcIcaOmaaqaeabaGaeyiLdq0aaSbaaSqaaiabdMgaPnaaCaaameqabaGaeGOmaidaaaWcbeaaaeqabeqdcqGHris5aOGaeiykaKYaaWbaaSqabeaacqaIXaqmcqGGVaWlcqaIYaGmaaGccqqGSaalcqqGGaaicqqHuoardaWgaaWcbaGaemyAaKgabeaakiabg2da9maalaaabaGaemOta40aaSbaaSqaaiabdkhaYjabdwgaLjabdggaHjabdYgaSnaaBaaameaacqWGPbqAaeqaaaWcbeaakiabgkHiTiabgYda8iabd6eaonaaBaaaleaacqWGYbGCcqWGHbqycqWGUbGBcqWGKbazdaWgaaadbaGaemyAaKgabeaaaSqabaGccqGH+aGpaeaacqWGobGtdaWgaaWcbaGaemOCaiNaemyzauMaemyyaeMaemiBaW2aaSbaaWqaaiabdMgaPbqabaaaleqaaOGaey4kaSIaeyipaWJaemOta40aaSbaaSqaaiabdkhaYjabdggaHjabd6gaUjabdsgaKnaaBaaameaacqWGPbqAaeqaaaWcbeaakiabg6da+iabgUcaRiabew7aLbaacqqGSaalcqqGGaaicqqGHbqycqqGUbGBcqqGKbazcqqGGaaicqaH1oqzcqGH9aqpcqaI0aanaaa@7985@. The criteria taken for enriched motifs are *N_real_Zscore *> 2, *N_real_*/*N_rand _*> 1.1, *Uniqueness *≥ 4 where Uniqueness is the number of times a motif appears in the network with completely disjoint groups of nodes [17,18].

To quantify the motif transitivity, we give the definition of motif transitivity score (MTS) as MTS=3×number of 'Δ' - number of 'V'3×number of 'Δ' + number of 'V'
 MathType@MTEF@5@5@+=feaafiart1ev1aaatCvAUfKttLearuWrP9MDH5MBPbIqV92AaeXatLxBI9gBaebbnrfifHhDYfgasaacH8akY=wiFfYdH8Gipec8Eeeu0xXdbba9frFj0=OqFfea0dXdd9vqai=hGuQ8kuc9pgc9s8qqaq=dirpe0xb9q8qiLsFr0=vr0=vr0dc8meaabaqaciGacaGaaeqabaqabeGadaaakeaacqWGnbqtcqWGubavcqWGtbWucqGH9aqpdaWcaaqaaiabiodaZiabgEna0kabb6gaUjabbwha1jabb2gaTjabbkgaIjabbwgaLjabbkhaYjabbccaGiabb+gaVjabbAgaMjabbccaGiabbEcaNiabfs5aejabbEcaNiabbccaGiabb2caTiabbccaGiabb6gaUjabbwha1jabb2gaTjabbkgaIjabbwgaLjabbkhaYjabbccaGiabb+gaVjabbAgaMjabbccaGiabbEcaNiabbAfawjabbEcaNaqaaiabiodaZiabgEna0kabb6gaUjabbwha1jabb2gaTjabbkgaIjabbwgaLjabbkhaYjabbccaGiabb+gaVjabbAgaMjabbccaGiabbEcaNiabfs5aejabbEcaNiabbccaGiabbUcaRiabbccaGiabb6gaUjabbwha1jabb2gaTjabbkgaIjabbwgaLjabbkhaYjabbccaGiabb+gaVjabbAgaMjabbccaGiabbEcaNiabbAfawjabbEcaNaaaaaa@7A19@, where 'Δ' is a group of 3 vertices each of which is connected to the other two, and 'V' is a group of 3 vertices only one of which is connected to the other two. The 'Δ' and 'V' are mutually exclusive subgroups in the MTS calculation. The factor of 3 accounts for the fact that each 'Δ' is equivalent to three 'V'. This formula quantifies the motif transitivity in the range from -1 to 1, and is *insensitive *to the motif size. The MTS is 1 for a fully connected motif, and is -1 for a motif without the triangle. The values of MTS for triads and tetrads are listed [see additional file 1, supp. table S1].

## Authors' contributions

PY designed and performed the study and drafted the manuscript. JSB helped conceive the study and guided the work. BDP and FAS helped contribute ideas discussed in the manuscript. All authors read and approved the final manuscript.

## Supplementary Material

Additional File 1 contains supplemental figures and tables. Click here for file
