# Supplementary material for: Commensurate distances and similar motifs in genetic congruence and protein interaction networks in yeast
Source: BMC Bioinformatics. 2005 Nov 9;6:270. doi: 10.1186/1471-2105-6-270 (PMC1310633; doi:10.1186/1471-2105-6-270)
Supplement: Additional File 1 — contains supplemental figures and tables. [file 1471-2105-6-270-S1.pdf]

Supplement for

Commensurate distances and similar motifs in genetic congruence and protein interaction networks in yeast

Ping Ye, Brian D. Peyser, Forrest A. Spencer, Joel S. Bader

Supp. Table S1. Motif transitivity score (MTS) for triad and tetrad motifs.

	Motif	$3\times$number of 'Δ'	number of 'V'	MTS
Triad1	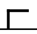	0	1	–1
Triad2	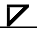	3	0	1
Tetra1	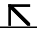	0	3	–1
Tetra2	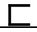	0	2	–1
Tetra3	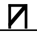	3	2	0.2
Tetra4	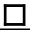	0	4	–1
Tetra5	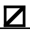	6	2	0.5
Tetra6	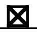	24	0	1

Supp. Table S2. The relative motif ratio (RMR) is insensitive to the threshold values for congruence and protein networks.

The indicated thresholds were used to define retained edges. Enriched motifs are triad2 and tetrad6 for both congruence networks. Enriched motifs are triad2, tetrad3 and tetrad6 for protein network with threshold 0.5, triad2 and tetrad6 for the network with threshold 0.65. The criteria for enriched motif are defined in [1] (see Methods).

		RMR			
		Symmetric congruence network		Protein network	
		Threshold = 6	Threshold = 8	Threshold = 0.5	Threshold = 0.65
Triad1	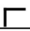	–0.70	–0.74	–0.29	–0.36
Triad2	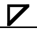	0.72	0.67	0.96	0.93
Tetra1	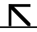	–0.57	0.32	–0.23	–0.09
Tetra2	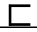	0.10	–0.10	–0.34	–0.44
Tetra3	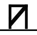	–0.23	–0.20	0.09	–0.18
Tetra4	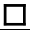	–0.33	0.00	–0.80	–0.67
Tetra5	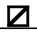	–0.67	–0.92	–0.14	–0.32
Tetra6	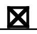	0.23	0.09	0.42	0.47

Supp. Table S3. Motif counts in the observed congruence network and in congruence networks obtained from 100 randomized synthetic lethal interaction networks.

Motif		Asymmetric congruence network			Symmetric congruence network		
		Observed	Random		Observed	Random	
		motif count	average motif count±s.d.	z-score	Motif count	average motif count±s.d.	z-score
Triad1		491	590.8±75.8	–1.3	151	657.6±47.4	–10.7
Triad2		435	26.6±3.4	118.9	267	64.8±10.4	19.3
Tetra1		232	687±279	–1.6	36	1142±194.5	–5.7
Tetra2		1211	1487.8±336	–0.8	236	2371.2±245.8	–8.7
Tetra3		1208	248.4±93.6	10.3	306	798±182.3	–2.7
Tetra4		29	8.8±3.9	5.2	3	57.8±16	–3.4
Tetra5		403	16±7.1	54.2	86	115.6±41.5	–0.7
Tetra6		693	0.4±0.5	1264.5	472	9±3.1	150.2

Randomizations were conducted as shown in Supp. Fig. S3. Transitive motifs triad2 (triangle) and tetrad6 (4-clique) have significantly higher counts in the observed network than the random networks. Intransitive motifs triad1, tetrad1, tetrad2, and tetrad4 have lower counts in the observed network than the random networks.

Supp. Table S4. Summary of *path length* comparisons between genetic/congruence networks and protein networks.

	Asymmetric genetic network	Symmetric genetic network	Asymmetric congruence network	Symmetric congruence network
Un-weighted protein network	Fig. 2A	Fig. 2A	Fig. 2D	Fig. 2D
Weighted protein network (edge weights correspond to interaction confidence)	Supp. Fig. S5A	Supp. Fig. S5A	Supp. Fig. S5C	Supp. Fig. S5C

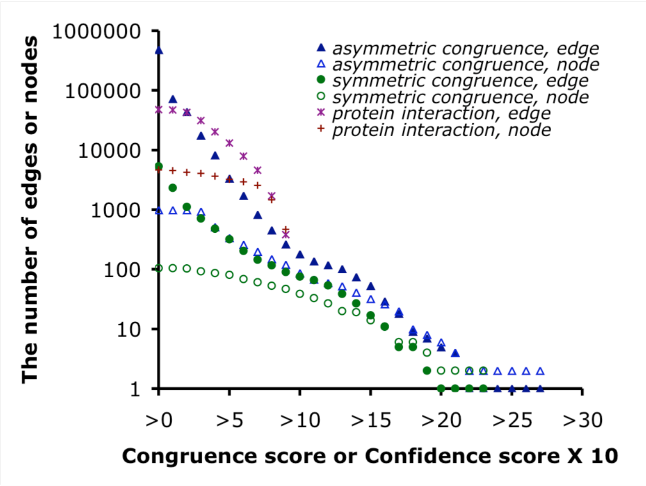

Supp. Fig. S1. The distribution of network size over different congruence scores or confidence scores for congruence and protein networks.

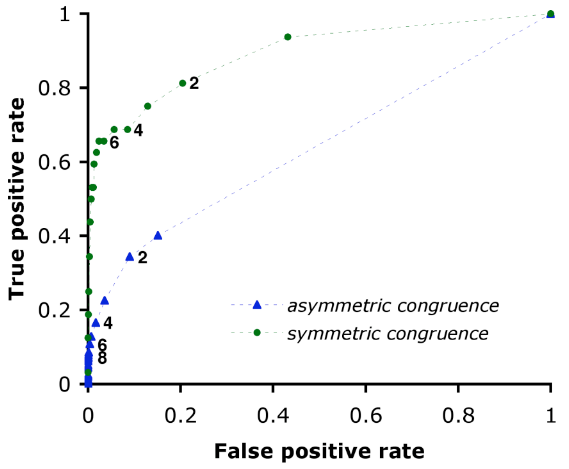

Supp. Fig. S2 - Prediction of high confidence protein interaction (with confidence score greater than 0.5 [2]) using congruence score is presented as a receiver operating characteristic (ROC) curve.

The numbers labeled next to the symbols are cut-off values for congruence scores.

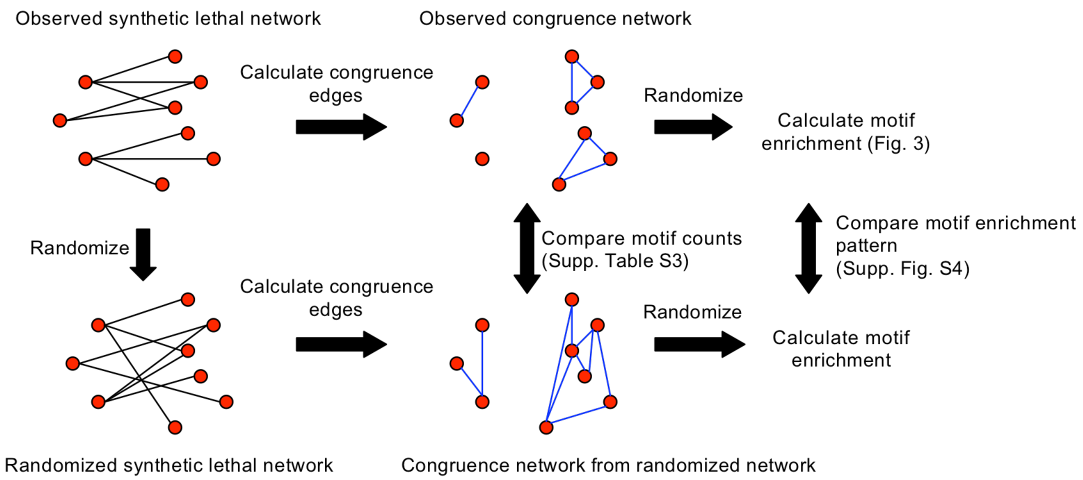

Supp. Fig. S3. Randomization scheme. The observed synthetic lethal interaction network was randomized 100 times keeping the mean number of interaction partners fixed for each gene, congruence scores were calculated for each of the 100 randomized networks, and congruence networks were constructed by choosing a threshold that yielded as many congruence edges as in the observed network. The mean thresholds were 3.2 (asymmetric) and 1.8 (symmetric), compared with 8 (asymmetric) and 6 (symmetric) for the observed congruence network. Motifs counts in the observed congruence network and the congruence network constructed from the randomized networks are compared in Supp. Table S3. The patterns of motif enrichment are compared in Supp. Fig. S4.

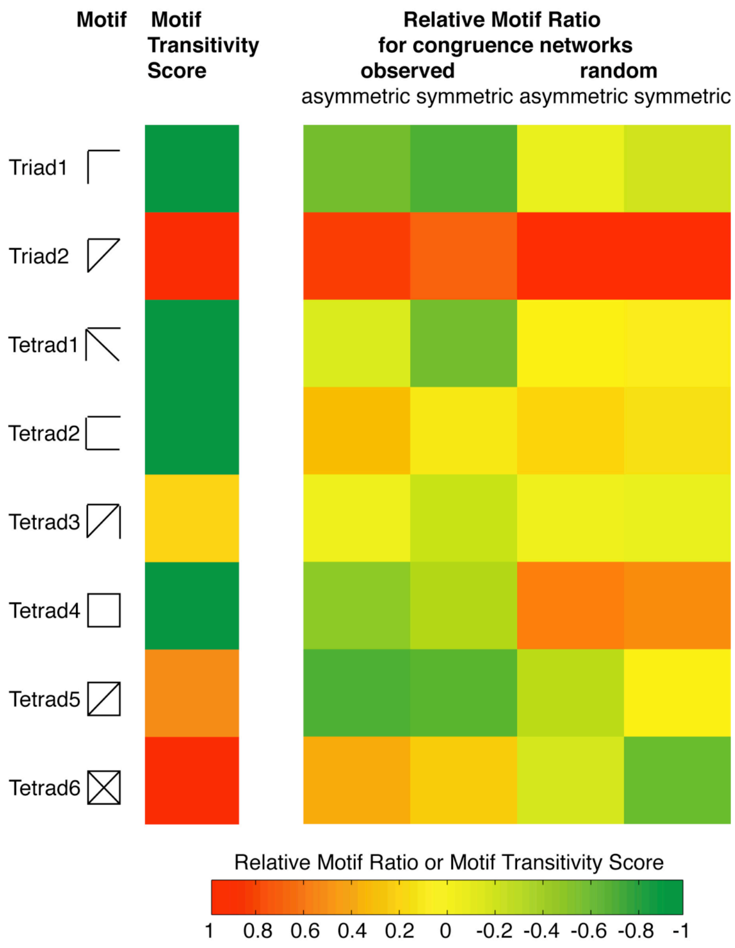

Supp. Fig. S4. Motif enrichment in congruence networks constructed from random networks. Congruence networks were constructed from a series of 100 randomized synthetic lethal interaction networks (see Supp. Fig. S3 and Supp. Table S3). The motif enrichment for the random networks displays a different pattern from the observed network. Although triad2 (triangle) is enriched in the observed and random networks, the raw number of triangles is far larger in the observed network (Supp. Table S3). The intransitive motif tetrad4 (square) is depleted in the observed network and enriched in the random networks, and the transitive motif tetrad6 (4-clique) is enriched in the observed network and depleted in the random networks.

A

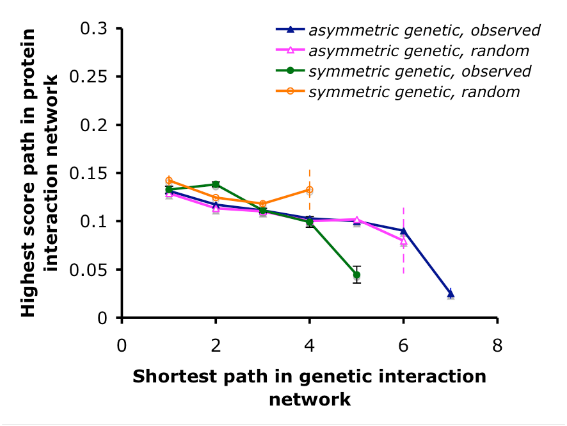

B

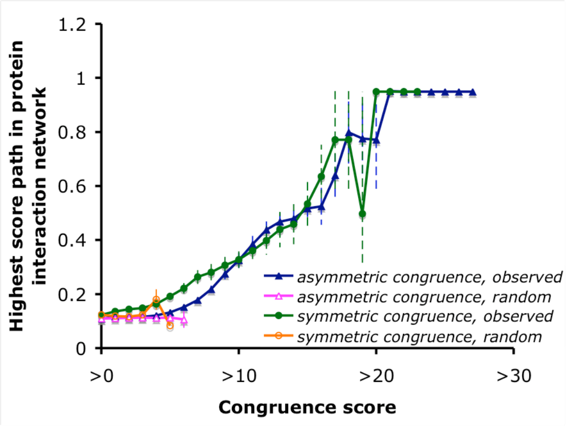

C

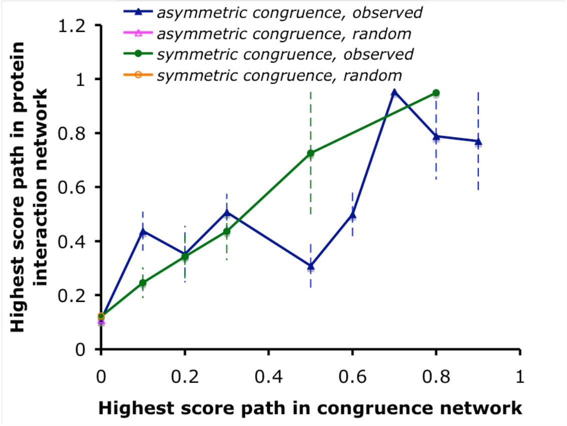

Supp. Fig. S5. By using highest score path distance in protein network, the path length is still incommensurate for genetic and protein networks but commensurate for congruence and protein networks.

A. The path distance in the protein network slightly declines with the corresponding distance in the genetic network (compare with Fig. 2A). **B**. The path distance in the protein network increases monotonically with congruence score (compare with Fig. 2C). **C**. The path distance in the protein network increases with the corresponding distance in the congruence network (compare with Fig. 2D). Results are displayed for the observed and randomized networks. Error bars indicate one standard error. The random value if present is comparable to the observed value (P-value > 0.05).

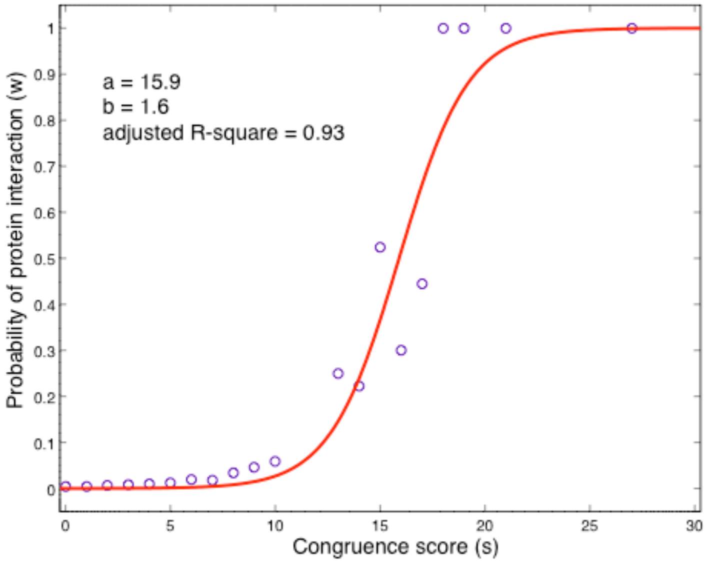

Supp. Fig. S6. The distribution of probability for protein interaction given the congruence score in the asymmetric congruence network.

The blue circles indicate the probability, which is fit with the sigmoid function $w = \frac{e^{(s-a)/b}}{1+e^{(s-a)/b}}$ represented by the red curve.

A

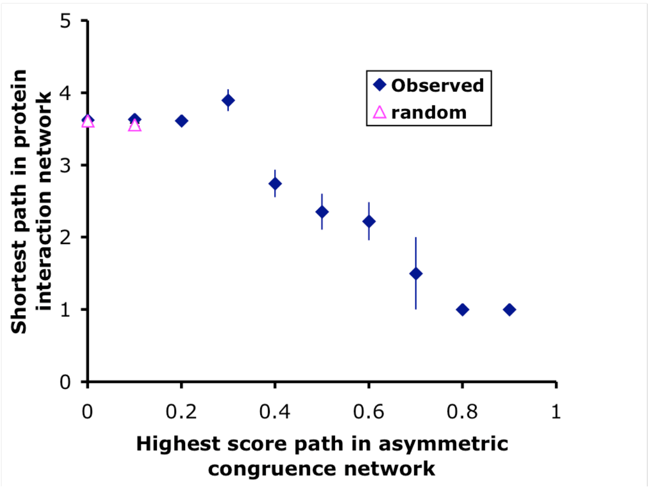

B

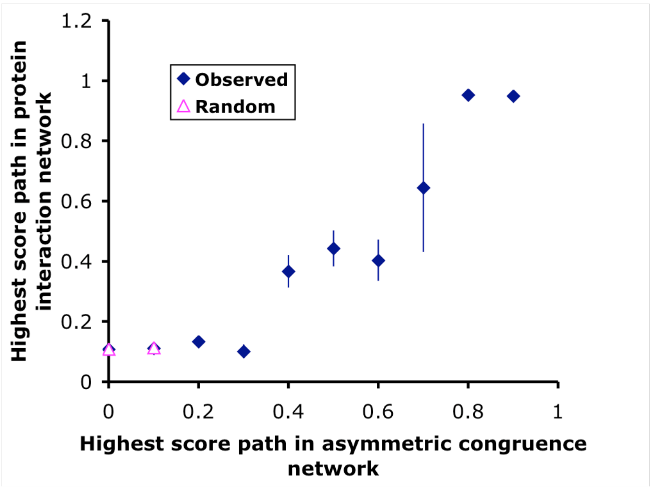

Supp. Fig. S7. The network distance results for the congruence network are not sensitive to the choice of parameter values for the sigmoid function $w = \frac{e^{(s-a)/b}}{1+e^{(s-a)/b}}$ **converting the congruence score to the edge weight ranging between 0 and 1.**

The values $a = 13.5$ and $b = 5$ were used for the distance calculation in the asymmetric congruence network instead of the best-fit values $a = 15.9$ and $b = 1.6$ (compare A with Figure 2D, and B with Figure S4C for asymmetric congruence network).

References

1. Milo R, Shen-Orr S, Itzkovitz S, Kashtan N, Chklovskii D, Alon U: **Network motifs: simple building blocks of complex networks**. *Science* 2002, **298**(5594):824-827.
2. Bader JS, Chaudhuri A, Rothberg JM, Chant J: **Gaining confidence in high-throughput protein interaction networks**. *Nat Biotechnol* 2004, **22**(1):78-85.
